# Programming Pluripotent Precursor Cells Derived from *Xenopus* Embryos to Generate Specific Tissues and Organs

**DOI:** 10.3390/genes1030413

**Published:** 2010-11-18

**Authors:** Annette Borchers, Tomas Pieler

**Affiliations:** Department of Developmental Biochemistry, Center of Molecular Physiology of the Brain (CMPB), GZMB, University of Goettingen, Justus-von-Liebig-Weg 11, 37077 Goettingen, Germany; E-Mail: tpieler@gwdg.de

**Keywords:** *Xenopus*, animal cap, pluripotent cells, organ development

## Abstract

*Xenopus* embryos provide a rich source of pluripotent cells that can be differentiated into functional organs. Since the molecular principles of vertebrate organogenesis appear to be conserved between *Xenopus* and mammals, this system can provide useful guidelines for the directional manipulation of human embryonic stem cells. Pluripotent *Xenopus* cells can be easily isolated from the animal pole of blastula stage *Xenopus* embryos. These so called “animal cap” cells represent prospective ectodermal cells, but give rise to endodermal, mesodermal and neuro-ectodermal derivatives if treated with the appropriate factors. These factors include evolutionary conserved modulators of the key developmental signal transduction pathways that can be supplied either by mRNA microinjection or direct application of recombinant proteins. This relatively simple system has added to our understanding of pancreas, liver, kidney, eye and heart development. In particular, recent studies have used animal cap cells to generate ectopic eyes and hearts, setting the stage for future work aimed at programming pluripotent cells for regenerative medicine.

## 1. Introduction

Amphibians have traditionally been used as model systems for vertebrate embryogenesis. They are particularly suited for the study of early embryonic patterning and induction events. In the 1920s, Hans Spemann and Hilde Mangold demonstrated that transplantation of a small piece of dorsal mesoderm (the “Spemann organizer”) into the ventral epidermis of a recipient embryo results in the formation of a complete secondary body axis [1]. This epochal work inspired generations of developmental biologists. However, the molecular control of this phenomenon has only been determined in the last two decades [2]. 

During the late 1960s, Peter Nieuwkoop and colleagues discovered that ectodermal cells can differentiate into mesodermal tissue if combined with prospective endodermal cells of the vegetal pole [3]. These so-called animal cap cells (ACC) can be isolated from the blastocoel roof of a blastula stage *Xenopus* embryo. If ACC are cultured without further manipulation they develop into “atypical epidermis”. Combination of ACC with vegetal cells generates dorsal, intermediate or ventral mesodermal tissue, depending on whether the vegetal fragment was taken from the dorsal or ventral side of the embryo [4]. Subsequent work demonstrated that ACC can also differentiate into neural or endodermal tissue. Neural induction can be achieved by inhibition of TGFß-signaling, thereby defining the neural fate as a default state [5], or by application of FGF [6]. Finally, ACC can also be turned into endoderm: In a cell-autonomous manner, VegT, a vegetally localizing, maternal T-box transcription factor, or its downstream transcription factor Mixer, can induce the expression of endodermal marker genes [7,8,9]. All of these studies using manipulated ACC were primarily based on the analysis of the activation of germ layer-/tissue-specific marker gene expression, as analyzed by either RT-PCR or whole mount *in situ* hybridization based techniques. 

Taking the use of the ACC system a step further, various higher order organotypic structures were generated employing a two-step protocol. First, ACC were programmed using different concentrations/combinations of regulatory molecules as outlined above. While the programming protocols utilized in these studies were mostly empirical, others studies have aimed to recapitulate the genetic program that controls *in vivo* organogenesis. Second, programmed ACC were transplanted into *Xenopus* embryos where they generated organ specific cell types or functional organs (see also the following paragraphs on individual organ systems, and for a recent review [10]). Striking examples are the generation of beating hearts or functional eyes from *Xenopus* ACC (see also below). Thus, the discovery of the ACC as a source for pluripotent precursor cells, which can be directed to differentiate into a broad variety of specialized cell types, has paved the road for their use in the generation of ectopic, functional organs *in vivo*.

## 2. Programming ACC to Generate Specific Tissues and Organs

### 2.1. The Experimental Basis

The pluripotent cells of the blastocoel roof, the ACC, are organized in several ectodermal cell layers, normally fated to develop into epidermis. They can be cultivated in simple saline solution for up to one or two weeks [11], corresponding to tadpole stages of development, when the primary organs have been formed. Manipulation of the corresponding explants can be performed in various ways:

a) ACC can be exposed to extracellular signaling molecules like growth factors or chemical agents affecting signal transduction. Recombinant growth factors can be applied for short intervals or throughout the incubation time. Alternatively, growth factors can be produced by the ACC themselves. To this end, fertilized embryos are injected with mRNA or DNA coding for the respective growth factor prior to dissection of the ACC.
b) Intracellularly active proteins, such as transcription factors, signal transduction proteins, *etc*. can also be supplied by microinjection of the corresponding mRNAs. As a variation of this procedure, microinjected explants can be sandwiched with naïve ACC, allowing for the distinction of cell-autonomous and non-cell-autonomous effects.
c) The developmental potential of specific tissues can be analyzed by heterotopic “Sandwich” assays combining ACC with tissue explants from the same or different embryonic sources.
d) Manipulated ACC can be transplanted into recipient embryos to analyze if they can form functional ectopic organs.

These different experimental protocols have not only been applied to the analysis of tissue/organ formation, but quite extensively also to problems relating to signal transduction mechanisms, cell adhesion phenomena and more. In addition to the activities employed in the pioneering studies mentioned above, various other factors, including secreted modulators and transcription factors, have successfully been used to direct ACC development. As outlined below, activin and retinoic acid, as well as the BMP-inhibitor noggin, have proven particularly useful to generate a broad range of tissue types from ACC. Furthermore, transcription factors can not only be used to generate a germ layer specific ground state (such as VegT for the endoderm), but combinations of transcription factors can drive organ specific development with high efficiency and specificity. An example of this is the generation of functional eyes by the eye field transcription factors. These and further examples of use of the ACC to induce different organs will be summarized in the following chapter.

### 2.2. Pancreas and Liver Development

**Figure 1:**
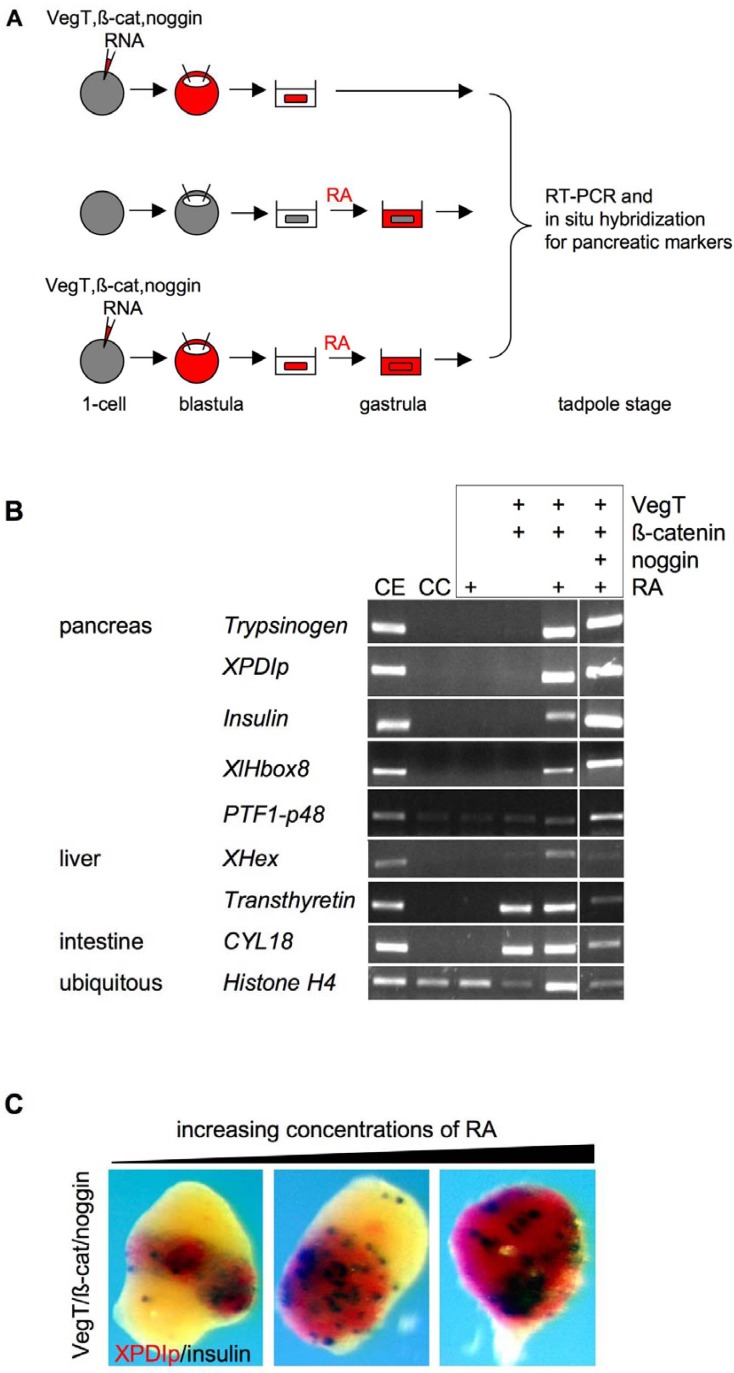
Induction of pancreas marker expression from pluripotent ACC. (**A**) ACC were induced either by injecting one-cell stage embryos with mRNAs coding for VegT, ß-catenin or noggin (top panel), or by incubating dissected ACC in retinoic acid (RA) (middle panel), or a combination of both (bottom panel). ACC were dissected at blastula stage and RA treatment was performed at late gastrula stage. Treated ACC were cultured until the equivalent of tadpole stages, when pancreatic marker expression was analyzed by RT-PCR and whole mount *in situ* hybridization; (**B**) RA induces pancreatic marker expression in ACC injected with *VegT* and *ß-catenin* mRNA. Additional injection of *noggin* mRNA leads to an increase in pancreatic marker expression. RT-PCR analysis of pancreas, liver and intestine marker gene expression of ACC treated as described in (A) (figure modified from [15]); CE, control embryo; CC, control ACC; (**C**) Increasing concentrations of RA (8, 16 or 32 µM) expand pancreatic marker expression in ACC injected with *VegT*, *ß-catenin* and *noggin* mRNA. XPDIp (red, marks exocrine pancreas) and insulin (blue, marks endocrine pancreas) expression was analyzed by whole mount *in situ* hybridization.

The vertebrate pancreas and liver are organs that play a key role in energy metabolism and homeostasis. They both originate from the anterior foregut endoderm, where the pancreas derives from a dorsal and a ventral anlage. The liver and ventral pancreas originate from a common population of precursor cells. High levels of FGF and BMP signaling from the adjacent mesodermal tissue induce the liver specific gene program and suppress pancreas specification. Additionally, retinoic acid (RA) signaling has a conserved, essential role for pancreas development (for a recent review, see [12]). The first success in the generation of pancreatic tissue from ACC came from the use of a two-step protocol, where the explants were first treated with activin and after its removal with RA [13]. The pancreatic tissue generated contained both endocrine (insulin and glucagon positive cells) as well as exocrine (acinus-like structures) components. In an attempt to more closely recapitulate early developmental control of pancreas formation, ACC were subsequently programmed with VegT, the key maternal transcription factor for formation of the endodermal germ layer. VegT alone was sufficient to induce the onset of a liver specific gene program, but addition of RA to the system proved essential and sufficient to induce both endocrine and exocrine pancreas-specific gene activity (Figure 1A, B). Additional application of a BMP-inhibitor was found to inhibit liver gene expression, thereby increasing the yield of pancreatic cells, which is consistent with the *in vivo* signaling situation [14,15,16] (Figure 1A, B). Indeed, at the appropriate concentration of RA, the majority of cells exhibited either endo- or exocrine pancreatic character (Figure 1C). Using *Xenopus* embryo microinjection, Afelik *et al.* [17] were able to demonstrate that ectopic expression of a combination of only two transcription factors, namely Pdx1 and Ptf1a, is sufficient to transform posterior endoderm into pancreas. Attempts to circumvent the need for RA in respect to pancreas induction in ACC, by applying VegT, Pdx1 and Ptf1a, were unsuccessful so far [18]. 

Due to the obvious medical interest in the generation of insulin-producing pancreatic ß-cells *in vitro*, numerous attempts using mammalian embryonic stem cells have been described in the literature (for recent reviews see [19,20]). Protocols for the generation of ß-cells from mammalian ES cells or from induced pluripotent stem cells (iPSCs), designed with the idea of recapitulating the relevant regulatory mechanisms as they operate during embryogenesis, have incorporated elements of the experiments using *Xenopus* ACC. In addition to other agents, including small molecules, activin and RA were successfully employed in the generation of pancreatic tissue from murine ES cells *in vitro* [21].

### 2.3. Kidney Development

The pronephros constitutes the primary secretory organ of *Xenopus* embryos. It is a derivative of the intermediate mesoderm and requires inducing signals to specify the pronephric anlagen during early gastrula stages of development. At tailbud stage, the developing pronephros becomes morphologically distinguishable and turns into a functional secretory organ (reviewed in [22]).

It was again Asashima and colleagues who first reported that pronephric structures can be generated from ACC *in vitro*. Histological analysis showed that ACC treated with a combination of activin and RA developed into pronephric tubules at a high frequency [23]. With the advent of pronephros-specific antibodies and molecular probes for pronephros-specific transcription activity, these initial observations were confirmed by several additional studies [24,25,26]. Strikingly, transplantation of these programmed ACC into *Xenopus* embryos, which had undergone bilateral pronephrectomy, suggests that they develop into functional secretory units. Although pronephrectomized embryos exhibited edema formation at high frequency and died after 7–9 days of surgery, transplanted animals formed less edema and had an increased survival rate [27]. 

A recent, elegant study, using a “sandwich” explant system including programmed ACC, identified a prime candidate signaling molecule for pronephros induction [28]. ACC were programmed with Wnt11 or Wnt11b by means of mRNA microinjection and used to form conjugate explants with intermediate mesoderm dissected from early gastrula stage *Xenopus* embryos. The programmed ACC or the intermediate mesoderm cultured in isolation did not reveal any significant indication of pronephros formation; however, their combination (“Holtfreter sandwich”) resulted in the formation of pronephric structures at high frequency. This procedure takes *in vitro* organ formation a step further by mimicking aspects of the often very complex tissue interactions.

### 2.4. Heart Development

The development of the heart is highly conserved among vertebrates. In *Xenopus*, heart development is initiated at the onset of gastrulation and requires an interplay of signals from the dorsal lip and the underlying endoderm. Initially, two patches of the mesoderm on the dorsal side of the embryo are specified towards cardiac fate. During gastrulation, these heart progenitors move dorso-anteriorly and continue to migrate ventrally at neurula stages. After meeting at the ventral midline, they fuse and form the linear heart tube, which then undergoes the looping and remodeling processes of cardiac morphogenesis.

Analyzing the precise molecular events that determine cardiac fate and ultimately formation of the heart is a topic of intense ongoing research. *Xenopus* provides a unique combination of straightforward gain- and loss-of-function analyses with a remarkable ability to heal after microsurgery, which has enabled *Xenopus* to become one of the favorite model systems for studying cardiogenesis [29,30]. In a landmark study, Horst Grunz exploited these advantages and generated beating heart muscle tissue *in vitro* by treating dorsal lip explants with suramin, a polyanionic compound [31]. Transplantation of these suramin-treated explants into the posterior trunk area of early tailbud embryos led to the formation of ectopic beating heart structures [32]. These experiments set the stage for *Xenopus* explants as valuable tools for the molecular analysis of cardiac induction and differentiation.

**Figure 2:**
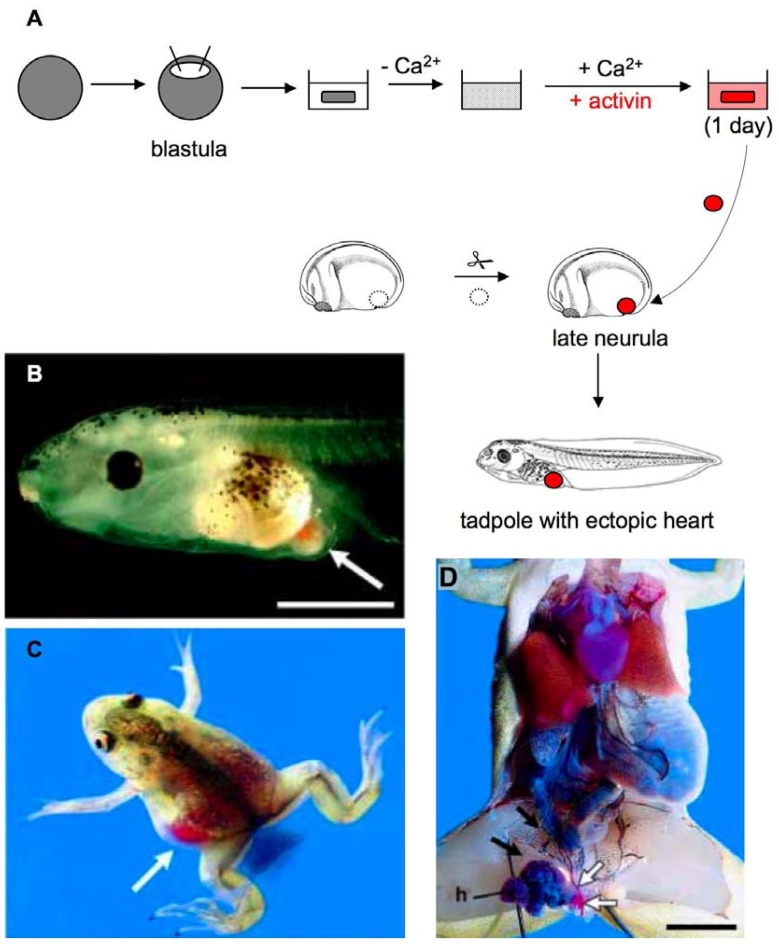
Generation of ectopic hearts by transplantation of *in vitro* induced ACC. (**A**) ACC were dissected at the blastula stage and dissociated by culturing in calcium-free medium. Dissociated cells were reaggregated by incubation in a saline solution containing calcium and activin. A fragment of the reassociated ACC was transplanted into the posterior abdomen of a neurula stage host embryo and developed to an ectopic beating heart at tadpole stage; (**B**) Tadpole embryo with a beating ectopic heart (white arrow); (**C**) Young frog displaying an ectopic *in vitro* induced heart. The ectopic heart is situated in the left abdomen and is filled with red blood cells (white arrow); (**D**) Internal anatomy of a one-year old frog with an ectopic heart (h) adjacent to the host's intestine. The blood flow from the host’s mesenteric artery (black arrow) to the anterior abdominal vein (white arrow) via the ectopic heart is indicated. (B–D) were reproduced from [37] with permission from The International Journal of Developmental Biology (*Int. J. Dev. Biol.* **2003**, *47*, 405–410). Scale bars: (B) 1 mm, (D) 5 mm.

In particular, ACC have proven to be a powerful and resourceful system to analyze the regulation of cardiogenesis. Using ACC, numerous transcription factors like GATA4, members of the Sox family or secreted signaling molecules like Wnt11 have been shown to induce cardiac marker expression [33,34,35]. The frequent use of ACC for the analysis of cardiogenesis started with the observation that activin-treatment of newt ACC induced a beating heart-like phenotype [36]. Asashima and colleagues succeeded in developing a method that induced cardiomyocytes at a high frequency [37] (Figure 2). ACC were dissociated by the removal of calcium and reassociated using a calcium- and activin-containing medium (Figure 2A). Reaggregated cells began beating at the same time as heart beating is initiated in embryos. In order to analyze if the *in vitro*-generated myocardial cells were functional, activin-treated ACC reaggregates were implanted into the posterior abdomen of neurula stage control embryos. Ectopic beating hearts were generated (Figure 2B–D), which consisted of at least two chambers and were involved in the systemic circulation. Since the first usage of ACC by Asashima and colleagues, this assay system has been extensively used as well as further modified. It has been demonstrated that injection of femtomolar concentrations of activin-encoding mRNA can replace the dissociation step [38]. Further, conjugation of ACC with anterior endoderm has also been shown to lead to cardiac induction, thus allowing analysis of the molecular contribution of endoderm to this process. Using this strategy, Samuel *et al.* [39] defined the time windows where FGF, Nodal and Wnt/ß-catenin signaling affect cardiogenesis. Their data demonstrate that FGF and Nodal signaling are required for cardiogenesis, while Wnt/ß-catenin signaling antagonizes cardiac differentiation. Thus, ACC proved to be a valuable tool for characterizing the molecular events involved in cardiac specification. Further use of this system for research may contribute to an understanding of congenital heart disorders and to developing therapies for cardiac disease.

### 2.5. Eye Development

The vertebrate eye detects incoming light and converts it into electrical signals; this strikingly complex structure poses particular challenges for regenerative medicine. Not only are eye tissues, like the retina, composed of an intricate array of different cell types, but embryonic eye development is also achieved by a sequential induction process that is difficult to recapitulate *in vitro*. Asahima and colleagues succeeded in inducing eye development *in vitro* at a high frequency by combining animal caps with dorsal lip and lateral marginal zone cells [40]. The *in vitro* generated eyes were morphologically similar to normal eyes and extended and connected to the optic tectum if transplanted into host embryos. Furthermore, if the *in vitro* induced eyes were transplanted into tadpole embryos, where both endogenous eyes had been removed, the resulting frogs could adapt their skin color to the color intensity of their background, indicating that the eyes were functional. These experiments showed that pluripotent ACC can be used to reproduce eye development *in vitro* and set the stage for the identification of the molecular factors involved in this process. 

The generation of retinal precursor cells is of particular interest, as it could provide an opportunity to treat retinal injuries or degeneration. Recently, factors like the BMP inhibitor noggin, or a set of genes commonly called the eye field transcription factors, have been shown to be able to drive pluripotent ACC towards a retinal fate. Lan *et al.* [41] showed that high concentrations of noggin, a secreted molecule that generates neural tissue by inhibition of BMP signaling, is sufficient to elicit retinal fates in animal caps. Noggin-injected animal caps cultured to tadpole stages at which embryonic retinogenesis is almost complete, expressed terminal differentiation markers of specific retinal cell types. Interestingly, the cell distribution was reminiscent of the retina lamination suggesting that noggin-injected animal caps display a basic retinal patterning. Furthermore, if the presumptive eye field of an embryo was replaced with an isochronic animal cap expressing a high concentration of noggin, the transplanted ACC formed a complete eye with a lens. Moreover, light-induced c-fos expression and electrophysiological characterization of the single-rod photoreceptors strongly suggest the functionality of the animal-cap-derived eye. 

**Figure 3:**
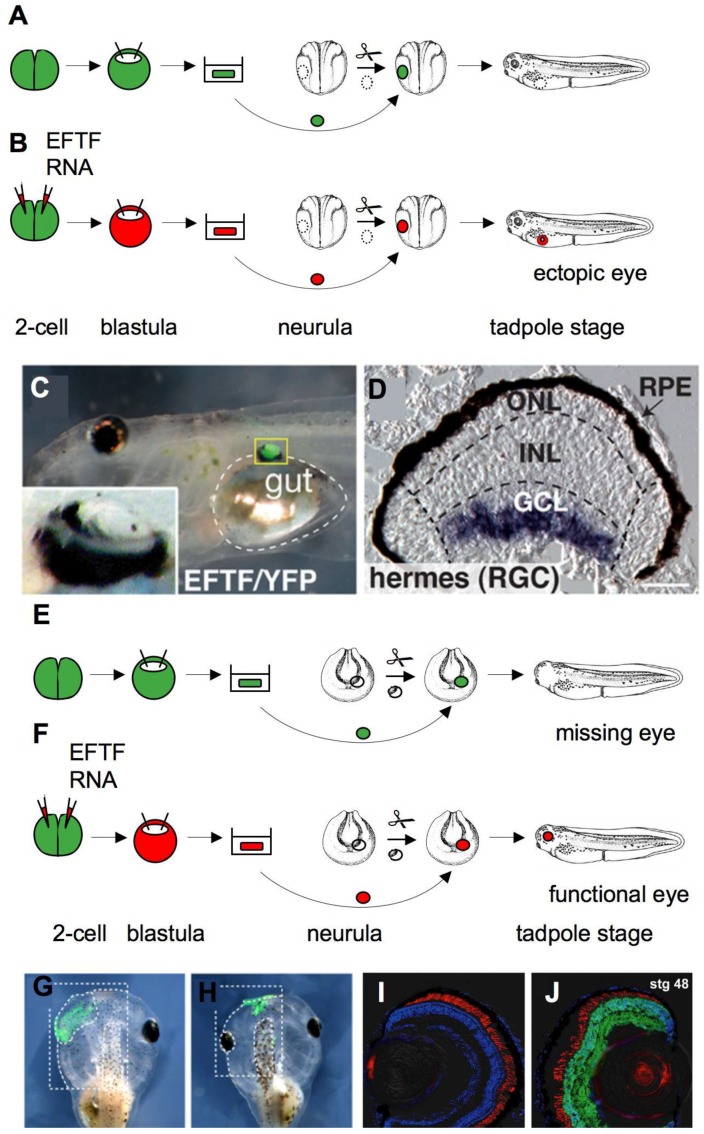
*In vitro* induction of functional eyes from ACC. (**A**) ACC were dissected from blastula stage transgenic embryos constitutively expressing yellow fluorescent protein (YFP). When transplanted into the flanks of neurula stage embryos, these ACC developed into sheets of epidermis; (**B**) In contrast, ACC injected with mRNA coding for a set of seven different transcription factors, collectively called the eye field transcription factors (EFTF), generated ectopic eyes after transplantation; (**C**) Tadpole embryo with ectopic EFTF/YFP-expressing eye in close proximity to the gut (indicated by dashed lines). Inlay shows a magnification of the ectopic eye; (**D**) *In situ* hybridization showing the expression of the retinal ganglion cell (RGC) marker, hermes, in a section of the ectopic eye. Outer nuclear (ONL), inner nuclear (INL) and ganglion cell layers (GCL) are indicated; RPE, retinal pigment epithelium; (**E**) Half of an animal cap from an uninjected transgenic embryo is not sufficient to generate an eye, if it is used to replace portions of the eye field; (**F**) In contrast, half of an animal cap expressing EFTF is able to replace the eye field and generate a functional eye. Control ACC expressing YFP only form epidermis (**G**), while EFTF-expressing ACC generate an eye (**H**); Insert in (H) shows a higher magnification of the eye. Sections of the control (**I**) and induced eye (**J**) of the same tadpole embryo. YFP fluorescence indicates that the eye originates from donor tissue. Figures (C,D and G–J) were reproduced from a PLoS Biology article by Viczian *et al.* [42]. Scale bar: (D) 12 µm.

An inspiring recent study demonstrated that ACC overexpressing a combination of seven transcription factors, the so-called eye field transcription factors (EFTF) and the neural patterning gene otx2, differentiate into all retinal cell classes and form a neuronal network necessary for vision [42]. Using an extended microarray analysis Viczian *et al.* showed that EFTF-expressing pluripotent ACC share a common transcriptional profile with the eye field. EFTF-expressing cells gave rise to ectopic eyes when they were transplanted into the flanks of a control embryo (Figure 3A–D), indicating that they are determined to a retinal fate. However, electrophysiological characterization was not possible, because these eyes were small and could not connect to their normal tectal targets in this ectopic location. Thus, the authors grafted EFTF-expressing animal caps into the eye field of host embryos (Figure 3E–J). These transplanted ACC generated all seven classes of differentiated retinal cells observed in the normal retina and showed a stereotypical retinal cell morphology, suggesting that these eyes are functionally equivalent to normal eyes. This was supported by the finding that electroretinograms of eyes, exclusively derived from EFTF-expressing ACC, were similar to those of eyes from the unoperated side of the same animal. The authors even went one step further and used phototropic behavior tests to confirm eye functionality. Interestingly, tadpoles with induced eyes showed the same vision-dependent preference towards a white background as normal embryos, confirming that these eyes originating from manipulated ACC were indeed functional. 

Taken together, these data show that pluripotent ACC can form diverse retinal cell classes and even functional eyes, if treated with the appropriate combination of factors. Thus, these studies open the way to characterize the role of various intrinsic and extrinsic factors and their combinations for their ability to direct pluripotent ACC to a retinal fate. Future challenges will be to determine how to maintain cultures of retinal precursor cells and to analyze if they could be used for retinal repair of damaged or diseased eye tissue. 

## 3. Conclusions

The *Xenopus* ACC system has already a long and successful tradition, but it is expected that it will further benefit from recent genetic and genomic advances. The use of mutant and transgenic *Xenopus* lines, which are increasingly available, remain to be exploited in this context. For example, the generation of transgenes carrying inducible versions of transcription factors would allow temporal control of their activation. Thus, more refined, multistep differentiation protocols could be developed. Once *Xenopus* lines with mutations in disease-associated genes are available, *in vitro* organ generation from such animals may be suitable for drug development. Moreover, advanced technologies that are based upon genomic sequence information, like microarray analysis and proteomics, are now readily available. Organ-specific tissues generated from manipulated ACC making use of wild-type as well as mutant *Xenopus* lines hold the promise of the discovery of novel genes and a more complete definition of the gene networks that govern organ development in vertebrates.

Several of the experiments outlined here were designed with the strategy to recapitulate key regulatory steps in organ development as they are known from embryological studies, others were equally successful using a more empirical approach. The most impressive example for the latter scenario is provided by the pioneering studies of the Asashima group, where different concentrations of RA in combination with activin were found to be sufficient to generate a whole variety of different organs and tissues [10]. These include pancreatic tissue, where RA has a conserved, essential signaling function during vertebrate embryogenesis. While this may also be the case for the development of the kidney [43], this is not clear yet for other organs. The use of combinations of eye field specific transcription factors is certainly the most rational strategy of mimicking the highly specialized situation characteristic of a given group of organ precursor cells. However, it remains to be shown if this strategy can also be applied to other types of organ precursor cells. 

*Xenopus* ACC define a source of pluripotent vertebrate precursor cells; their versatility is comparable to other populations of pluripotent precursor cells, such as mammalian IPS cells. A hope that is connected to the IPS system is that these pluripotent cells could be used to engineer patient-specific tissue or even organs for regenerative medicine. In general, the underlying molecular principles in vertebrate organogenesis appear to be highly conserved. Therefore, protocols established in the very robust—and therefore easy to handle—ACC system can serve as guidelines for the directional manipulation of mammalian embryonic stem cells.

Thus, looking back on half a century of exciting research with *Xenopus* animal cap explants makes us optimistic for another 50 years of groundbreaking insights into the secrets of organ development.

## References

[1] Spemann H. (1936). Experimentelle Beiträge zu einer Theorie der Entwicklung.

[2] De Robertis E.M. (2006). Spemann’s organizer and self-regulation in amphibian embryos. Nat. Rev. Mol. Cell Biol..

[3] Nieuwkoop P.D. (1963). Pattern Formation in Artificially Activated Ectoderm (Rana Pipiens and Ambystoma Punctatum). Dev. Biol..

[4] Dale L., Slack J.M. (1987). Regional specification within the mesoderm of early embryos of *Xenopus laevis*. Development.

[5] Hemmati-Brivanlou A., Melton D.A. (1992). A truncated activin receptor inhibits mesoderm induction and formation of axial structures in *Xenopus* embryos. Nature.

[6] Lamb T.M., Harland R.M. (1995). Fibroblast growth factor is a direct neural inducer, which combined with noggin generates anterior-posterior neural pattern. Development.

[7] Clements D., Woodland H.R. (2003). VegT induces endoderm by a self-limiting mechanism and by changing the competence of cells to respond to TGF-beta signals. Dev. Biol..

[8] Henry G.L., Melton D.A. (1998). Mixer, a homeobox gene required for endoderm development. Science.

[9] Horb M.E., Thomsen G.H. (1997). A vegetally localized T-box transcription factor in *Xenopus* eggs specifies mesoderm and endoderm and is essential for embryonic mesoderm formation. Development.

[10] Asashima M., Ito Y., Chan T., Michiue T., Nakanishi M., Suzuki K., Hitachi K., Okabayashi K., Kondow A., Ariizumi T. (2009). *In vitro* organogenesis from undifferentiated cells in *Xenopus*. Dev. Dyn..

[11] Fukui Y., Furue M., Myoishi Y., Sato J.D., Okamoto T., Asashima M. (2003). Long-term culture of *Xenopus* presumptive ectoderm in a nutrient-supplemented culture medium. Dev. Growth Differ..

[12] Zaret K.S., Grompe M. (2008). Generation and regeneration of cells of the liver and pancreas. Science.

[13] Moriya N., Komazaki S., Takahashi S., Yokota C., Asashima M. (2000). *In vitro* pancreas formation from *Xenopus* ectoderm treated with activin and retinoic acid. Dev. Growth Differ..

[14] Chen Y., Jurgens K., Hollemann T., Claussen M., Ramadori G., Pieler T. (2003). Cell-autonomous and signal-dependent expression of liver and intestine marker genes in pluripotent precursor cells from *Xenopus* embryos. Mech. Dev..

[15] Chen Y., Pan F.C., Brandes N., Afelik S., Solter M., Pieler T. (2004). Retinoic acid signaling is essential for pancreas development and promotes endocrine at the expense of exocrine cell differentiation in *Xenopus*. Dev. Biol..

[16] Pan F.C., Chen Y., Loeber J., Henningfeld K., Pieler T. (2006). I-SceI meganuclease-mediated transgenesis in *Xenopus*. Dev. Dyn..

[17] Afelik S., Chen Y., Pieler T. (2006). Combined ectopic expression of Pdx1 and Ptf1a/p48 results in the stable conversion of posterior endoderm into endocrine and exocrine pancreatic tissue. Genes Dev..

[18] Pan F.C., Afelik S., Pieler T. (2006).

[19] Borowiak M., Melton D.A. (2009). How to make beta cells?. Curr. Opin. Cell Biol..

[20] Mayhew C.N., Wells J.M. (2009). Converting human pluripotent stem cells into beta-cells: Recent advances and future challenges. Curr. Opin. Organ. Transplant.

[21] Nakanishi M., Hamazaki T.S., Komazaki S., Okochi H., Asashima M. (2007). Pancreatic tissue formation from murine embryonic stem cells *in vitro*. Differentiation.

[22] Brandli A.W. (1999). Towards a molecular anatomy of the *Xenopus* pronephric kidney. Int. J. Dev. Biol..

[23] Moriya N., Uchiyama H., Asashima M. (1993). Induction of Pronephric Tubules by Activin and Retinoic Acid in Presumptive Ectoderm of *Xenopus laevis*. Dev. Growth Differ..

[24] Heller N., Brandli A.W. (1999). *Xenopus* Pax-2/5/8 orthologues: novel insights into Pax gene evolution and identification of Pax-8 as the earliest marker for otic and pronephric cell lineages. Dev. Genet..

[25] Osafune K., Nishinakamura R., Komazaki S., Asashima M. (2002). *In vitro* induction of the pronephric duct in *Xenopus* explants. Dev. Growth Differ..

[26] Eid S.R., Terrettaz A., Nagata K., Brandli A.W. (2002). Embryonic expression of *Xenopus* SGLT-1L, a novel member of the solute carrier family 5 (SLC5), is confined to tubules of the pronephric kidney. Int. J. Dev. Biol..

[27] Chan T.C., Ariizumi T., Asashima M. (1999). A model system for organ engineering: transplantation of *in vitro* induced embryonic kidney. Naturwissenschaften.

[28] Tetelin S., Jones E.A. *Xenopus* Wnt11b is identified as a potential pronephric inducer. Dev. Dyn..

[29] Afouda B.A., Hoppler S. (2009). *Xenopus* explants as an experimental model system for studying heart development. Trends Cardiovasc. Med..

[30] Warkman A.S., Krieg P.A. (2007). Xenopus as a model system for vertebrate heart development. Semin. Cell Dev. Biol..

[31] Grunz H. (1992). Suramin changes the fate of Spemann’s organizer and prevents neural induction in *Xenopus laevis*. Mech. Dev..

[32] Grunz H. (1999). Amphibian embryos as a model system for organ engineering: *in vitro* induction and rescue of the heart anlage. Int. J. Dev. Biol..

[33] Latinkic B.V., Kotecha S., Mohun T.J. (2003). Induction of cardiomyocytes by GATA4 in *Xenopus* ectodermal explants. Development.

[34] Pandur P., Lasche M., Eisenberg L.M., Kuhl M. (2002). Wnt-11 activation of a non-canonical Wnt signalling pathway is required for cardiogenesis. Nature.

[35] Zhang C., Basta T., Klymkowsky M.W. (2005). SOX7 and SOX18 are essential for cardiogenesis in *Xenopus*. Dev. Dyn..

[36] Ariizumi T., Komazaki S., Asashima M., Malacinski G.M. (1996). Activin treated urodele ectoderm: A model experimental system for cardiogenesis. Int. J. Dev. Biol..

[37] Ariizumi T., Kinoshita M., Yokota C., Takano K., Fukuda K., Moriyama N., Malacinski G.M., Asashima M. (2003). Amphibian *in vitro* heart induction: A simple and reliable model for the study of vertebrate cardiac development. Int. J. Dev. Biol..

[38] Afouda B.A., Martin J., Liu F., Ciau-Uitz A., Patient R., Hoppler S. (2008). GATA transcription factors integrate Wnt signalling during heart development. Development.

[39] Samuel L.J., Latinkic B.V. (2009). Early activation of FGF and nodal pathways mediates cardiac specification independently of Wnt/beta-catenin signaling. PLoS One.

[40] Sedohara A., Komazaki S., Asashima M. (2003). *In vitro* induction and transplantation of eye during early *Xenopus* development. Dev. Growth Differ..

[41] Lan L., Vitobello A., Bertacchi M., Cremisi F., Vignali R., Andreazzoli M., Demontis G.C., Barsacchi G., Casarosa S. (2009). Noggin elicits retinal fate in *Xenopus* animal cap embryonic stem cells. Stem Cells.

[42] Viczian A.S., Solessio E.C., Lyou Y., Zuber M.E. (2009). Generation of functional eyes from pluripotent cells. PLoS Biol..

[43] Cartry J., Nichane M., Ribes V., Colas A., Riou J.F., Pieler T., Dolle P., Bellefroid E.J., Umbhauer M. (2006). Retinoic acid signalling is required for specification of pronephric cell fate. Dev. Biol..

